# Mechanistic Modeling of Zwitterionic Surfactant Adsorption on Mineral Surfaces: A Three-Step Isotherm with Micelle-Induced Desorption

**DOI:** 10.1021/acs.langmuir.5c02879

**Published:** 2025-08-26

**Authors:** Pablo A. Godoy, Luis Maqueira, Aurora Pérez-Gramatges

**Affiliations:** † Department of Chemistry, Pontifical Catholic University of Rio de Janeiro (PUC-Rio), Rio de Janeiro 22451-900, Brazil; ‡ Laboratory of Physical-Chemistry of Surfactants (LASURF), Pontifical Catholic University of Rio de Janeiro (PUC-Rio), Rio de Janeiro 22451-900, Brazil

## Abstract

Most studies on surfactant adsorption onto minerals have primarily relied on simplified isotherm models, which fail to account for the anomalous behavior observed after micelle formation. In this work, we introduce a novel three-step adsorption model that builds upon the classical two-step theory by incorporating a micelle-triggered desorption mechanism. This discontinuous and implicit step, activated at the critical micelle concentration, accounts for surface aggregate desorption induced by micelles, a phenomenon previously unaddressed in adsorption isotherms. The model was validated using experimental data for cocamidopropyl betaine adsorbing on both sandstone and limestone. The added mechanism is grounded in electrical double layer properties obtained via a Surface Complexation Model (SCM). It was found that surfactant adsorption is more favored in sandstone (2.15 mg/m^2^ of maximum adsorption) than limestone (1.85 mg/m^2^) due to headgroup affinity and higher packing onto adsorption sites. The proposed three-step model is in excellent agreement with our experimental data (adj-*R* ^2^ closer to unity) but also with previous data in the literature for zwitterionic and anionic surfactants. We demonstrated that the adsorption maximum is a consequence of aggregate desorption induced by micelles when surfactant head groups have the same charged nature as the solid–liquid interface. SCM simulations implied that this effect can be minimized by a heterogeneous distribution of charges on the rock surface. Lastly, the three-step model exponents can indicate extreme adsorption scenarios for higher concentrations, allowing estimations of long-term surfactant loss in the porous media.

## Graphical abstract

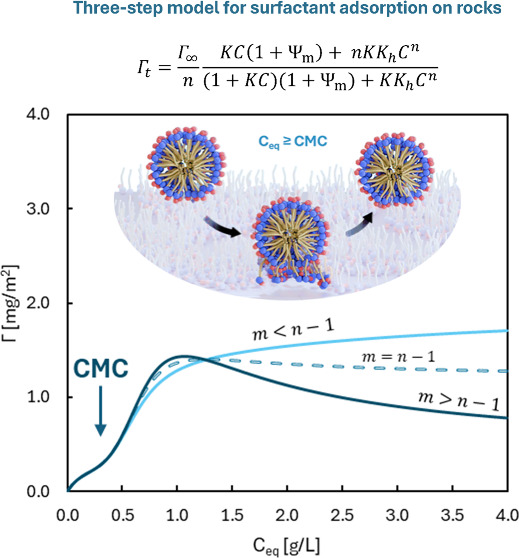

## Introduction

Surfactant technology in enhanced oil recovery (EOR) and geological carbon storage (GCS) is paramount to improve sweep efficiency and serve as a foundation for large-scale CO_2_ sequestration.^1–3^ Successful EOR pilots involving the injection of surfactants have been conducted on reservoirs with silicate and carbonate formations.^4–7^ However, the techno-economic feasibility of these subsurface operations relies largely on surfactant adsorption in the porous media, as it directly impacts operation costs and, in the case of foam technology, its generation and stability.^8–11^

Zwitterionic surfactants have gained attention in recent years as effective chemical additives for subsurface operations due to their interfacial properties, chemical stability after longer exposures to high reservoir temperatures and high salinity, and lower adsorption on carbonate formations.^12,13^ Cocamidopropyl betaine (CAPB), a large-produced carboxy amido betaine derived from coconut oil fatty acids, has been reported as a suitable zwitterionic surfactant for subsurface applications because of its low price, biodegradability, low toxicity to marine life, and strong foaming ability under harsh reservoir conditions.^14–16^

Few studies have addressed models to describe CAPB adsorption behavior on sandstone or limestone minerals and even fewer have established possible mechanisms beyond monomer-only adsorption.^17–19^ In adsorption studies conducted on sandstone under high temperature and salinity conditions, Dai et al. observed a Langmuir-type isotherm for CAPB adsorption. Similarly, Zhong et al. applied the Langmuir isotherm to model CAPB adsorption on shale rock in the presence of formation water. Although these works had led to Langmuir isotherms to explain the adsorption behavior of CAPB in seawater, no other work has investigated cooperative adsorption and micellar exclusion with a modeling approach for this surfactant. Yet, previous zeta potential results from Durán-Alvarez et al. indicated micellar exclusion after reaching critical micelle concentration (CMC) in an adsorption study using CAPB, which was attributed to charge buildup at the interface. Also, their molecular dynamics simulations revealed aggregate structures resulting from cooperative adsorption onto the mineral surface.^18^

Cooperative adsorption models are found in the literature to describe general surfactant adsorption behavior, accounting for surface aggregation because of lateral interactions.^20–22^ The one-step model proposed by Gu and Zhu was one of the first to address such behavior for surfactants. This model assumed that only surface aggregates by lateral interactions, i.e., hemimicelles, participate in surfactant adsorption/desorption equilibrium, creating an S-type isotherm. Later, both Zhu and Gu derived the two-step model incorporating monomer adsorption as a step prior to hemimicelle adsorption, leading to L–S type isotherms. Most recently, Zaafouri et al. developed a similar model to the two-step model in which hemimicelle kinetics is altered by the already adsorbed aggregate via a cooperative mechanism. Both the two-step and Zaafouri et al. frameworks could be used to include surface aggregation as a mechanism for CAPB adsorption.

Micellar exclusion is another mechanism that influences adsorption equilibrium, which leads to negative adsorption, or a decrease in adsorbed surfactant at concentrations above the CMC, generating a maximum in the isotherm curve.^18,23^ This mechanism creates dependence of the CMC value in adsorption, and such dependence was already incorporated in previous studies into the modeling of adsorption behavior for surfactants and mixtures.^24,25^ However, to our knowledge, no adsorption isotherm has yet been proposed that simultaneously accounts for surface aggregation and micelle influence on desorption despite reports of such mechanisms.^18^ This fact demands an extension of the previous theories for a more general adsorption behavior for any surfactant-mineral system that exhibits similar properties.

In this study, we develop a general three-step adsorption model to describe the adsorption behavior of the zwitterionic surfactant CAPB on rocks of varying mineralogy, extending the classical two-step theory by incorporating a third, CMC-dependent step. The model is validated using static adsorption experiments with CAPB dissolved in seawater and two representative rock types (sandstone and limestone) as adsorbents. Surfactant concentrations before and after adsorption were quantified using liquid chromatography. Additionally, adsorption data from the literature of other zwitterionic and anionic surfactants were used to further test the model applicability. To support the proposed mechanisms, adsorption trends are also correlated with surface species distributions and zeta potential estimations derived from a validated Surface Complexation Model (SCM).^26,27^

## Materials and Methods

### Chemicals and Rock Samples

The surfactant used in this work was a commercial formulation (Oxitaine CP 30 APH) from Oxiteno (Brazil), containing cocamidopropyl betaine (CAPB) as the active compound (30 wt %) and NaCl (approximately 5.5 wt %). The molecular structure is represented in Figure S1 in the Supporting Information. A synthetic desulfated seawater (DSW) was prepared by dissolving inorganic salts in ultrapure water (Milli-Q Direct 8 system, 18.2 MΩ cm at 25 °C) at the following composition: NaCl (27.865 g L^–1^), CaCl_2_ 2H_2_O (0.485 g L^–1^), MgCl_2_ 6H_2_O (1.270 g L^–1^), KCl (0.750 g L^–1^), NaHCO_3_ (0.099 g L^–1^), and NaSO_4_ (0.060 g L^–1^), to obtain 0.518 mol L^–1^ of ionic strength and 30.529 g L^–1^ of salinity. The pH and conductivity of the seawater were 7.95 and 45.55 mS cm^–1^, respectively. All salts were reagent grade from Sigma Aldrich, Brazil (>99% purity). Acetonitrile and Ammonium acetate from Supelco (HPLC grade) and Acetic Acid from Sigma Aldrich, Brazil (>99% purity), were utilized in the chromatographic method for the detection and quantification of CAPB. Pyrene for CMC determination was purchased from Sigma Aldrich and purified by sublimation.

Two rock plug samples, namely, Berea sandstone (silicate rock) and Indiana limestone (carbonate rock), were purchased from Kocurek Inc. The main mineralogy is quartz (88%), feldspar (5%), and kaolinite (4%) for Berea sandstone, and mostly calcite (98%) for Indiana limestone (Table S1 in Supporting Information).^27–33^

### Experimental Procedures

#### Rock Preparation and Specific Surface Area (SSA) Characterization

The rock powder used for static adsorption experiments was prepared by first crushing a piece of Berea sandstone and Indiana limestone core samples using a jaw crusher, followed by further trituration with a disc crusher. To ensure a homogeneous distribution of rock particles for the experiments, the powder was sieved through an electromagnetic sieve with 32 and 150 MESH sizes to obtain particles with a diameter of 111 μm or smaller. The solid sample was then cleaned using a Soxhlet apparatus, where toluene and then methanol were cycled through the powder for 24 h. Finally, the powder was dried in a vacuum oven at 90 °C and 0.1 bar for 24 h.

The fine powder of both rocks used as adsorbent material was analyzed using a TriStar II 3020 gas adsorption system with nitrogen. The specific surface area (SSA) was determined from the gas adsorption and desorption data using a Brunauer–Emmett–Teller (BET) isotherm adjustment, while the average micropore diameter was calculated using Barrett–Joyner–Halenda (BJH) analysis.

### Adsorption Experiments

The static adsorption of CAPB on rock powder was evaluated through batch experiments in which 1 g of rock powder was suspended in a 5 mL surfactant solution and shaken at 300 rpm and 30 °C using an orbital shaker with temperature control. All experiments were conducted for 24 h to allow the system to reach equilibrium. This time was chosen based on preliminary adsorption tests and information from literature.^17,19^ Aliquots of the suspension were collected with a syringe and filtered through a 0.22 μm micropore filter to remove any suspended rock particles. The initial (*C*_0_) and equilibrium (*C*_eq_) surfactant concentrations in the filtered solutions were determined by liquid chromatography and used to calculate the adsorbed amount (Γ). The experiments were performed in triplicate, with initial CAPB concentrations ranging from 0.10 to 1.00 g/L. Adsorption (mg/m^2^) was then calculated using eq 1, where the surfactant concentration is expressed in g/L, *V* is the volume of the solution in mL, *m* is the mass of the rock powder in grams, and SSA is the BET specific surface area of the rock in m^2^/g.

$$\Gamma =\frac{(C_{0}-C_{\mathrm{eq}})\times V}{m\times \mathrm{SSA}}$$ 1

### Surfactant Quantification

An Agilent 1260 Infinity II High-Performance Liquid Chromatography (HPLC) system equipped with a Diode Array Detector (DAD) from Agilent Technologies, USA, was used to detect and quantify the surfactant. Chromatographic separations were performed using an Acclaim Surfactant Plus column (250 × 4.6 mm, 5 μm) with a flow rate of 1.0 mL/min, an injection volume of 50 μL, and a column oven temperature of 30 °C. The detection wavelength was set to 215 nm. The mobile phase consisted of acetonitrile (eluent A) and a 0.1 M ammonium acetate buffer (pH 5.0, eluent B) prepared with ultrapure water (Milli-Q). Gradient elution was applied, starting with a ratio of 48% A to 52% B, increasing to 52% A over 20 min, and then held for 5 min before returning to the initial conditions. The system was equilibrated for 10 min before each sample injection. All chromatograms were recorded and analyzed using Agilent OpenLab CDS software (version 2.7). Figure S2 (Supporting Information) shows an example of the chromatographic profile of CAPB obtained under the described conditions.

### Critical Micelle Concentration Determination

Fluorescence spectroscopy was used to determine the critical micelle concentration (CMC) of the surfactant in DSW, with pyrene serving as the fluorescence probe.^34–38^ This technique allows for temperature control, thus matching conditions with adsorption experiments. Fluorescence measurements were conducted using a Cary Eclipse Fluorescence Spectrophotometer (Agilent Technologies, USA) at an excitation wavelength of 336 nm. The fluorescence emission spectra were recorded over the wavelength range of 360–450 nm at 30 °C, and the change in the 1:3 pyrene intensity ratio was used to identify the transition from monomers to micelles. The emission spectrum was normalized to the maximum intensity. The logarithm of the concentration and 1:3 pyrene ratio data were fitted using a Boltzmann regression (Figure S3a,b) in the Supporting Information). The CMC value was then calculated with CMC = *x*_0_ + 2Δ*x*, following the method proposed by Aguiar et al. for ionic surfactants, with the 1:3 pyrene ratio curves fitted by a Boltzmann regression (eq S14).^37^

### Three-Step Adsorption Model

The proposed model assumes the first two steps of the two-step theory: a single surfactant monomer (*A*) adsorbing on the available site (*S*) (eq 2), and other *n*–1 monomers adsorbing in the populated site (*SA*) to form hemimicelles (*SA*_*n*_) (eq 3). The new third step accounts for *m* surfactants in micellar form (A*) approaching the hemimicelle site and creating micelles-like surface aggregate (*SA*_*n*_ *A*_m_^*^) (eq 4), followed by its desorption and exclusion to the bulk (*A*_*n*_ *A*_m_^*^), leaving the site available again (eq 5). These were assumed to take place on the surface of both sandstone and limestone rocks.

A+S⇄SA 2

$$(n-1)A+\mathrm{SA}⇄{\mathrm{SA}}_{n}$$ 3

$${\mathrm{SA}}_{n}+mA^{*}\to {\mathrm{SA}}_{n}A_{m}^{*}\;\mathrm{for}\;C\geq \mathrm{CMC}$$ 4

$${\mathrm{SA}}_{n}A_{m}^{*}\to A_{n}A_{m}^{*}+S\;\mathrm{for}\;C\geq \mathrm{CMC}$$ 5

Assuming the intermediate surface aggregate (SA_*n*_ *A*_m_^*^) exists for a very short time, the last two reactions (eqs 4 and 5) can be combined into one (eq 6).

$${\mathrm{SA}}_{n}+mA^{*}\to A_{n}A_{m}^{*}+S$$ 6

This proposed mechanism implies that surfactant molecules can desorb as monomers, as hemimicelles, and as a combination of hemimicelles and micelles. The last one (eq 6) occurs when the concentration exceeds the CMC, and its kinetics are represented by the rate equation:

$$v_{m}=k_{5}\Gamma_{h}(C-\mathrm{CMC}{)}^{m}H(C-\mathrm{CMC})$$ 7

The term *H*(*C*–CMC) is the Heaveside function that implies *v*_*m*_ equal to zero if *C* < CMC, and different from zero when *C* ≥ CMC, in agreement with the fact that such a mechanism would not exist at equilibrium concentrations lower than the CMC. The rate constant *k*_5_ refers to the micelle induced desorption process, and Γ_*h*_ is the amount of adsorbed hemimicelles.

To combine this mechanism (eq 7) with the already deducted mass-action models from the two-step theory (eqs S1 and S2), *v*_*m*_ is added as a negative contribution to the hemimicelle adsorption rate (eq S4). Equilibrium is assumed to take place in both modes of adsorption, so expressions for the monomer-only adsorption step (Γ_1_) and hemimicelle adsorption step (Γ_*h*_) can be derived from mass-action models. Also, the total surfactant concentration used in mass-action models (*C*) is reinterpreted as the surfactant equilibrium concentration when equilibrium is assumed. The remaining two-step theory assumptions are maintained as a means of constructing the third-step isotherm model shown in eq 8 (see detailed deduction in the Supporting Information).

$$\Gamma_{t}=\frac{\Gamma_{\infty }}{n}\frac{\mathrm{KC}(1+\Psi_{m})+nKK_{h}C^{n}}{(1+\mathrm{KC})(1+\Psi_{m})+KK_{h}C^{n}}$$ 8

where *C* is the surfactant equilibrium concentration, *K* is the adsorption equilibrium constant of the monomer adsorption step, and *K*_*h*_ is the hemimicelle adsorption equilibrium constant. The term Ψ_*m*_ = *K*_*m*_(*C*–CMC)^*m*^ *H*(*C*–CMC) is the micellar-induced desorption contribution, and the constant *K*_*m*_ is defined as the ratio of the hemimicelle desorption rate constant with micelle induction (*k*_5_) to that without induction (*k*_4_, see eq S2). The condition where *K*_*m*_ is sufficiently high implies there is more hemimicelle desorption due to micelle induction than without it, and a sufficiently small *K*_*m*_ means the opposite.

When *C* < CMC, Ψ_*m*_ = 0, and we get the generalized L–S isotherms from classical two-step theory, which comprehends Langmuir-type isotherms and S-type isotherms.^20^ Therefore, the proposed model (eq 8) will behave like a two-step model until it reaches the CMC.

The two-step model will be used as a benchmark for classical adsorption behavior because its isotherm is derived from classical mass-action models. Those models can separately result in the Langmuir isotherm by considering monomer-only adsorption, and in the classical Sips isotherm, considering only hemimicelle dynamics. Since the three-step model is built upon the previous model, it incorporates classical isotherm behavior and demonstrates the impact of the extra added step.

### Model-Fitting Method

The model fitting to adsorption data was implemented in MATLAB by utilizing the Non-Linear Least Squares method with the trust-region algorithm. Since we cannot know for sure if the set of converged parameters from the model is a global solution of the optimization, the fitting is performed hundreds of times with random variations upon the converged parameters (Figure S4 in Supporting Information).^39^ The result is obtained after several iterations, or if the change in the parameters is within the tolerance (0.1%). If the solutions lead to parameters located on the boundaries of the input parameter range, the boundary values would be changed to lay the converged value between the new ones. The procedure then repeats until the result parameter is a nonboundary value.

To evaluate the performance of the proposed model against the two-step model, the adjusted *R* ^2^ (adj-*R* ^2^) and the Root Mean Square Error (RMSE) were calculated after each fit. These metrics account for the degrees of freedom of each model (number of parameters), thus penalizing the ones with too many parameters, avoiding overfitting of the data, and allowing for model performance comparison.

### Surface Complexation Modeling

The Charge Distribution Multisite Complexation (CD-MUSIC) model^40–42^ was chosen to represent the electrical double layer (EDL) properties in both Berea sandstone/DSW and Indiana limestone/DSW interfaces, and to estimate the distribution of surface species according to the potentially determining ions (PDIs) present in the DSW. PHREEQC software was used to run the CD-MUSIC model. Temperature, pH, and composition of the DSW were inputs to the model alongside the BET specific surface area (SSA) of both adsorbents. All literature-adjusted parameters for quartz, kaolinite, and calcite were also included in the CD-MUSIC model (Table S2).^26,27^ The representative surface species for sandstone and limestone, as described in SCMs, include those found on minerals such as quartz, kaolinite, and calcite, like metal-hydroxyl groups (>SiOH, > AlOH, > CaOH) and carbonates (>CO_3_H).^26,27,41,43–45^ The site density of quartz and kaolinite was adjusted according to their reported fractions in the Berea sandstone composition (Table S3 in Supporting Information), since the total rock SSA is used as input, not the individual mineral surface areas. For Indiana limestone, the entire site density was attributed to calcite.

## Results and Discussion

### Static Adsorption of CAPB and Modeled Isotherms

To evaluate the proposed three-step adsorption model for CAPB behavior, static adsorption experiments were conducted using DSW as the aqueous phase and sandstone and limestone as the solid phases. The CMC of the surfactant in seawater, used as an input parameter in the model, was determined using the pyrene fluorescence method (Figure S3). The data were fitted using Boltzmann regression (eq S14), yielding a CMC value of 0.232 g/L.

The static adsorption profiles of the zwitterionic surfactant, expressed in mg/g of rock, were similar for both rocks at lower concentrations (below CMC value) (Figure 1a). When maximum adsorption was reached, Berea sandstone exhibited slightly higher adsorption values than limestone.

**Figure 1:**
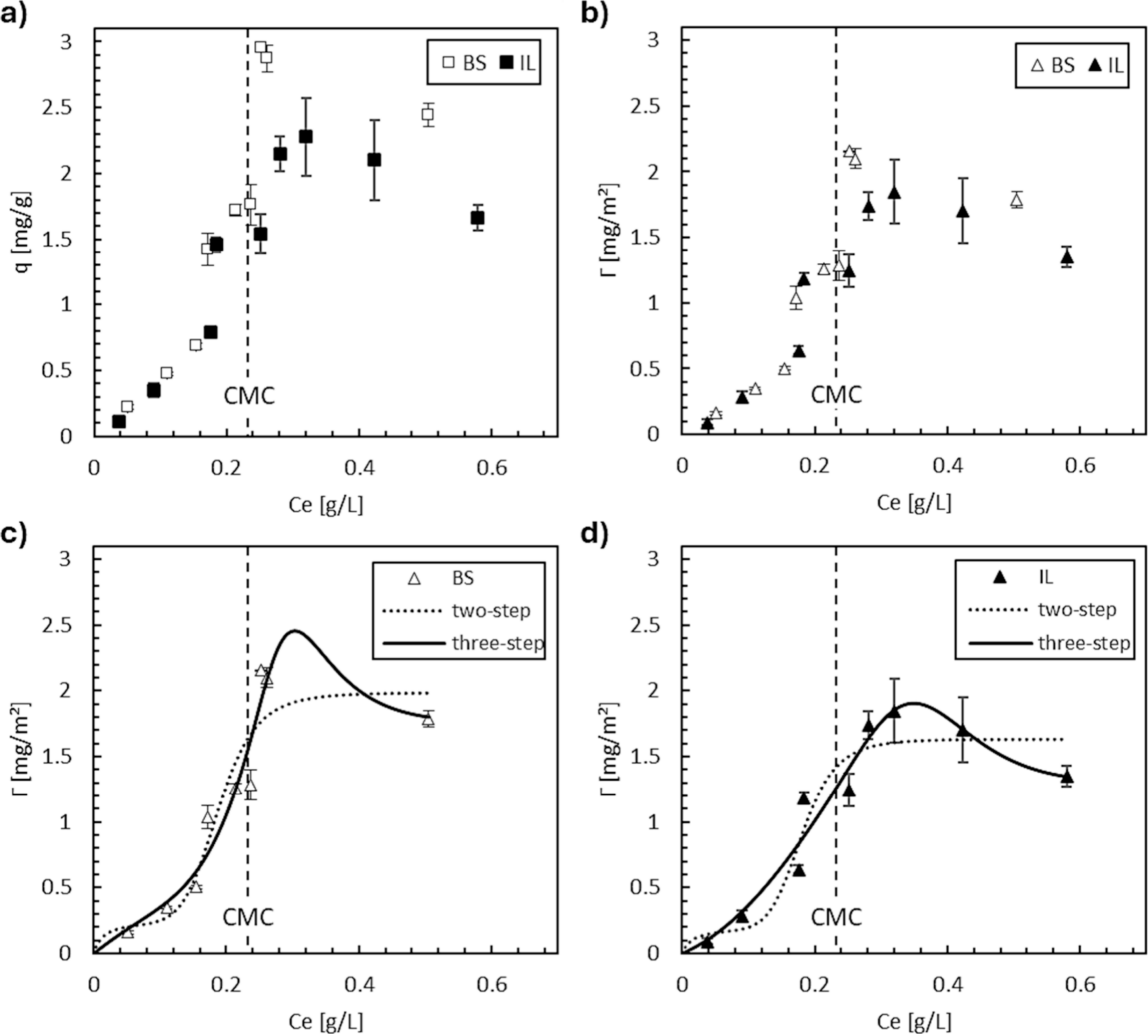
Adsorption profiles of the zwitterionic surfactant CAPB, (a) normalized by adsorbate mass (*q*, mg/g) and (b) by surface area (Γ, mg/m^2^), and the two-step (eq S13) and three-step (eq 8) modeled isotherms for adsorption on (c) Berea sandstone (BS) and (d) Indiana limestone (IL). CMC: critical micelle concentration of CAPB in seawater.

Given that sandstone and limestone had their granulometry up to 111 μm in diameter (maximum grain size), the total surface area available could be different due to different distributions below that size. Thus, the adsorption data were normalized using the specific surface area (SSA) values for each rock (Figure 1b). The normalization by SSA (1.37 m^2^/g for Berea sandstone and 1.23 m^2^/g for Indiana limestone) reduced both the overall adsorption magnitude and the differences between the rocks, effectively eliminating the impact of surface area on model fitting. Both the two-step and three-step models were fitted to experimental data (Figure 1c,d), and the three-step model developed in this work clearly shows the best fit.

Both adsorption profiles resemble S-type isotherms, indicating the formation of surface aggregates resulting from lateral interactions, which suggest a cooperative adsorption mechanism taking place under these conditions.^46,47^ The maximum CAPB adsorption per unit surface area was 2.15 mg/m^2^ for Berea sandstone and 1.85 mg/m^2^ for Indiana limestone. It is worth noting that the presence of positively and negatively charged groups in the molecular structure of the zwitterionic surfactant allows interactions with surface groups on both types of rocks.

### Adjusted Adsorption Parameters and Their Interpretation

The new three-step model parameters (eq 8) were compared with the classical two-step model (eq S13), since both share most of the parameters. Regarding performance, it is evident from RMSE and adj-*R* ^2^ values that the three-step model is more suited to predict CAPB adsorption on both rocks (Table 1).

**Table 1:** Fitting Results of Two-Step and Three-Step Models to CAPB Adsorption Data in Berea Sandstone and Indiana Limestone^a^

	Berea Sandstone		Indiana Limestone	
models	two-step	three-step	two-step	three-step
adj-*R* ^2^	0.81	0.84	0.84	0.87
RMSE	0.32	0.30	0.26	0.23
**Γ**_**∞**_ (mg/m^2^)	1.99	8.99	1.63	8.00
K (L/g)	69.9	2.20	65.4	0.64
K_h_ (L/g)^n‑1^	6.90 × 10^4^	111.4	1.62 × 10^5^	5.3
K_m_ (L/g)^m^	-	1684	-	423
n	7.64	4.91	7.97	2.21
m	-	3.01	-	3.34
CMC (g/L)	-	0.232	-	0.232

a For the interpretation of model parameters, see the Experimental Procedures section.

The adsorption of the zwitterionic surfactant is influenced by electrostatic and van der Waals interactions, which play an important role in the first step of adsorption, as well as by hydrophobic interactions, which will govern the second and third steps of adsorption behavior.^8,10,48^ The equilibrium constant values indicate the intensity of each step regarding monomer and hemimicelle adsorption or desorption.

Both the two-step and three-step models demonstrate that the CAPB monomer adsorption (first step) is more favorable on the sandstone, as evidenced by the higher values of *K*. However, in the two-step model, *K*_*h*_ is higher for limestone, indicating dominant hemimicelle adsorption (second step). This result does not align with the trend observed in the adsorption experiments (Figure 1a,b), nor with the reported tendency of zwitterionic surfactants to adsorb more favorably onto sandstone minerals, such as quartz and kaolinite, compared to carbonate surfaces like calcite or dolomite.^49–52^ The *K*_*h*_ values determined by the three-step model provide a better explanation of the differences in adsorption behavior, as the constant value for sandstone is nearly 20 times higher than for limestone. Generally, the *K*_*h*_ equilibrium constant will be lower in the three-step model than in the two-step model because of the extra pathway of hemimicelle desorption (micelle-induced). The lower *K*_*h*_ indicates reduced hemimicelle coverage at equilibrium in the three-step model, and vice versa.

The *K*_*m*_ constant, defined only in the three-step model, correlates well with the effect of micelles inducing hemimicelle desorption, where the sandstone holds its highest value. We used surface speciation and zeta potential results obtained from CD-MUSIC to explain why micelle-induced hemimicelle desorption is higher on sandstone. Since sandstone surface starts with predominantly neutral and negative adsorption sites (Figure 2a and Table S4), surfactants start to cover these sites by quaternary ammonium interactions, then the zeta potential becomes less negative, creating the possibility of micelles approaching the sites. This is the previous step to the migration of hemimicelles to micelles since the dominant hydrophobic interactions required are more effective at short range. However, the detailed mechanism at the molecular level by which this migration occurs has no impact on the mathematical assumptions for the proposed model. We leave the investigation of the molecular bases for future research involving molecular dynamics simulations.

**Figure 2:**
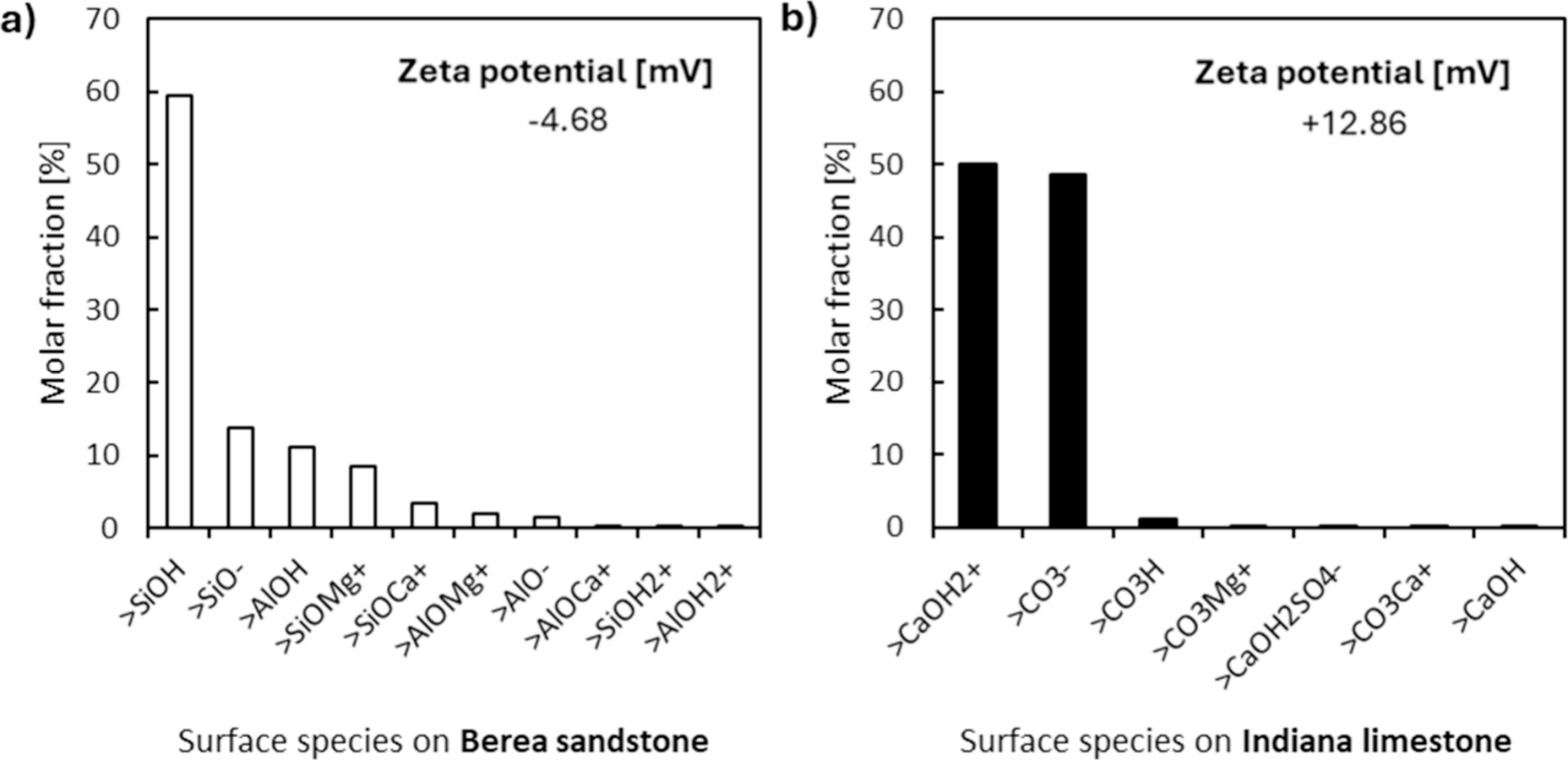
CD-MUSIC results for surface speciation and zeta potential estimations in (a) sandstone-seawater interface and (b) limestone-seawater interface.

Once micelles start to blend with hemimicelles, their negative moiety becomes exposed to negative sites, initiating their exclusion from the interface due to repulsion forces (Figure 3). The same mechanism occurs with limestone, but the effect has less intensity, since the micelle-hemimicelle aggregate is exposed to more positive charges on the rock surface, and it decreases the aggregate exclusion due to more attractive forces (Figures 2b and 3). The resulting *K*_*m*_ value for limestone is 1 order of magnitude lower than that for sandstone, supporting the previously stated hypothesis.

**Figure 3:**
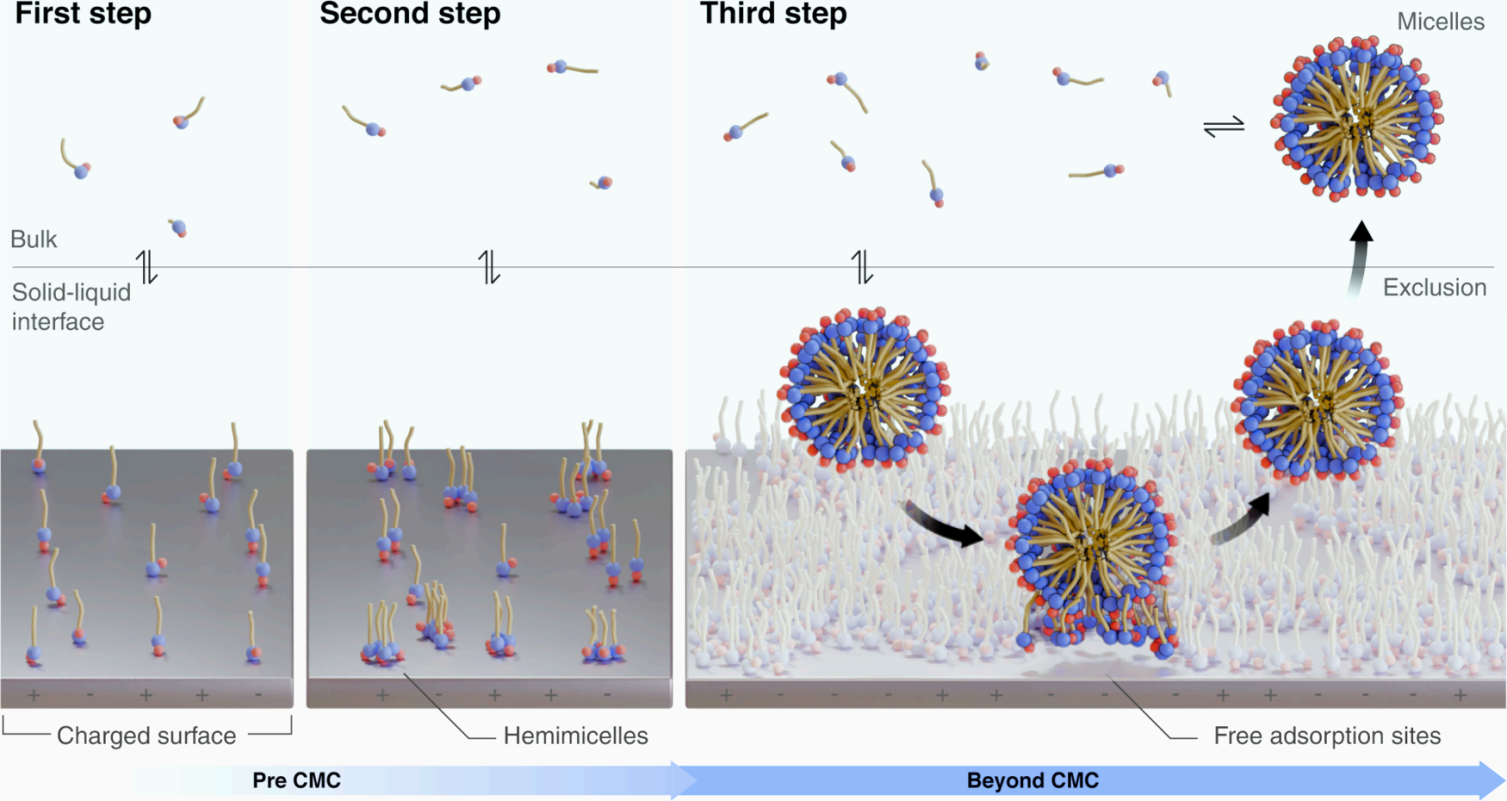
Schematics of the three-step model and the respective adsorption steps. In the third step, micellar-like aggregates are excluded from the rock surface due to electrostatic repulsion, while attraction forces between the negative surfactant headgroups and positive rock sites reduce micelle-induced desorption of hemimicelles.

The surface speciation estimations correlate with maximum adsorption capacity (Γ_∞_) since the CAPB molecules interact more with negative and neutral sites due to a favorable conformation,^50^ showing its highest value in the sandstone case. The classical two-step model underestimates the value of Γ_∞_ for both rocks, leading to the misleading conclusion that they have similar capacities and minimal differences at higher surfactant concentrations. However, the total amount of surfactants that can be packed on each surface differs significantly due to the distinct electrostatic nature of the mineral surfaces in seawater.

Exponent *n* has a greater value in the sandstone case since it accounts for the number of monomers in a hemimicelle. A larger *n* means better packing of the CAPB molecules on the sandstone surface.^51^ Because there are more CAPB molecules in one hemimicelle on sandstone than on limestone, fewer micelles are required to induce desorption, resulting in a lower *m* for adsorption in sandstone. Conversely, the two-step model leads to the conclusion that CAPB molecules are better packed on limestone as it presents higher *n*, which contradicts our surface speciation estimates, and the adsorption results reported in the literature for zwitterionic surfactants.^19,49,50,52^

### Model Convergence and Maximum Surface Capacity

One question that arises from analyzing the three-step expression (eq 8) is related to the total adsorption (Γ_*t*_), as we can keep increasing surfactant concentration until reaching considerably high values. It turns out that it can converge either to Γ_∞_ or Γ_∞_/*n* (see Supporting Information). The plateau value depends on a relationship between the parameters *m* (number of surfactants in micellar form inducing desorption) and *n*–1 (number of monomers adjacent to the first monomer to adsorb). The Γ_∞_/*n* ratio indicates a surface covered only with monomers, since hemimicelles are constantly being dismantled by micelles (Figure 4a, dark dashed line). Yet, a plateau on Γ_∞_ means that the surface is completely full of hemimicelles reaching maximum adsorption capacity, because micelles cannot induce hemimicelle desorption faster than their adsorption (Figure 4a, solid line).

**Figure 4:**
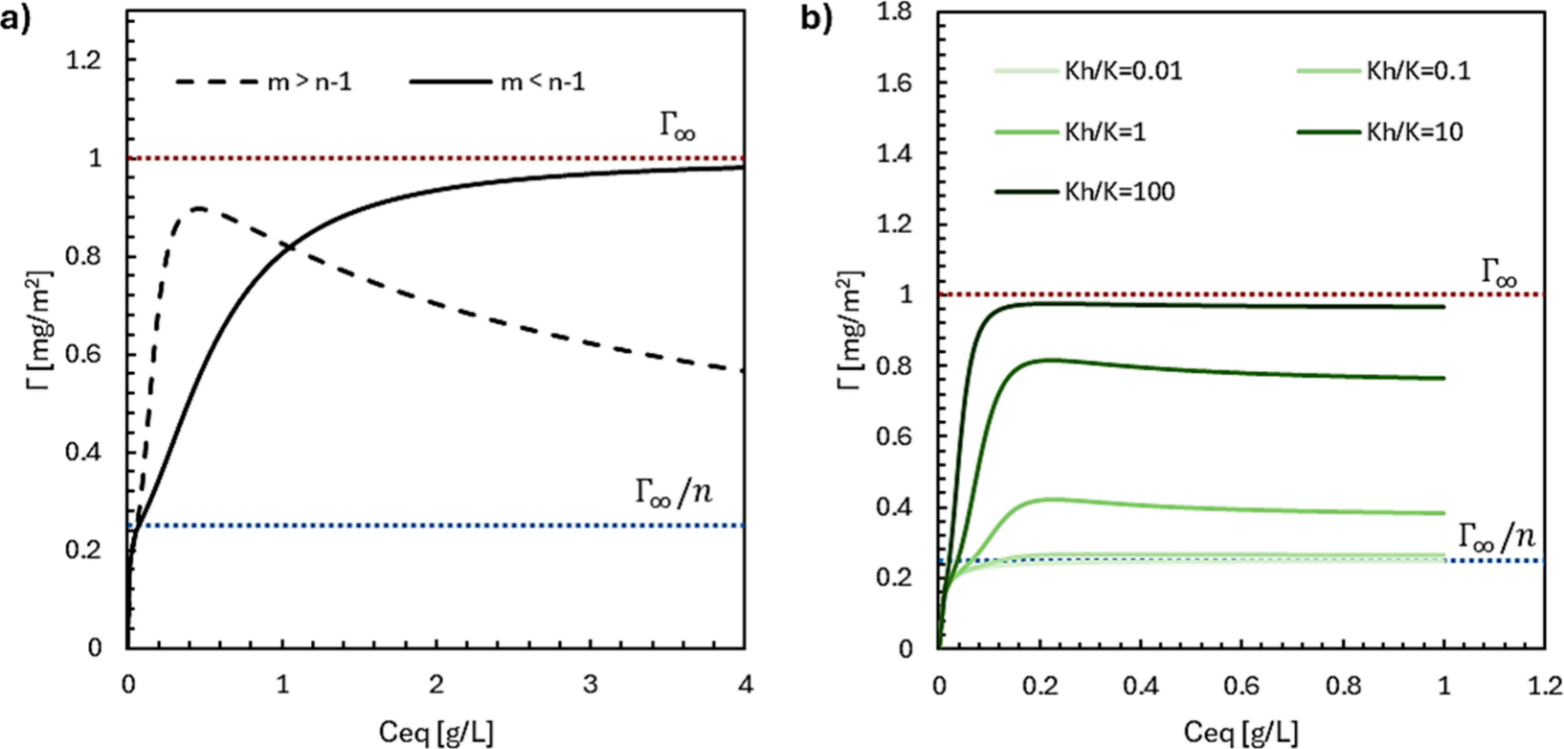
Illustrative convergence of the three-step model under different relationships between parameters. (a) *m* > *n*–1 (dashed line) and *m* < *n*–1 (solid line), with the adsorption plateaus when *C*_eq_ → ∞ (dotted lines). (b) *m* = *n*–1, for a range of *K*_*h*_/*K* ratios, where low ratios (brighter lines) are closer to L-type isotherms, and high ratios (darker lines) are closer to S-type isotherms. The parameters used in these plots were not representative of prior data.

There is a special case when *m* equals *n*–1: the ratio between hemimicelle (*K*_*h*_) and monomer (*K*) equilibrium constants controls which value the plateau will be closer to (Figure 4b). The scenarios alternate between a surface saturated with hemimicelles (Γ_∞_) when *K*_*h*_/*K* > 1, and a surface with one monomer per site (Γ_∞_/*n*) when *K*_*h*_/*K* < 1. These limits approximate reality as, statistically, it is highly improbable that numbers will match exactly, but when they get closer, the plateau could indicate a surface covered with both monomers and hemimicelles in equilibrium.

The relationships *m* > *n*–1 (for any *K*_*h*_/*K*) and *m* = *n*–1 (for *K*_*h*_/*K* < 1) demonstrate the persistence of an L-shape isotherm in a sufficiently wide range of concentrations (Figure 4a,b). This observation aligns with prior studies of CAPB adsorption behavior, which reported Langmuir-type isotherms.^17,19^ These literature findings could be interpreted as an approximation of our proposed model at higher concentrations, where the cooperative behavior of adsorbed molecules is compromised by micelles, resulting in an apparent single-monomer adsorption.

These results are particularly valuable for planning surfactant applications in subsurface operations, where concentrations typically exceed the CMC. In such a case, reaching the *m* < *n*–1 condition is undesirable, as it results in the highest possible surfactant adsorption onto reservoir rock, increasing long-term surfactant loss. The main advantage of determining the three-step model parameters is to assess the *m* and *n* parameters prior to the operation, to identify the conditions for minimum surfactant adsorption to occur. This approach enables the use of surfactant concentrations above the CMC while minimizing adsorption at the rock–seawater interface, particularly when the model indicates micelle-induced desorption.

### Discontinuity in Adsorption Isotherms

The idea of using the CMC as a point of discontinuity on static adsorption isotherms is not new.^53,54^ Previous works had attributed micelle influence in the monomer desorption; however, the three-step model proposed in this work is the first isotherm model to account for the micelle influence in the desorption of surface aggregates. It implements that idea in a mechanistic manner, which improves the classic two-step model and performs better fittings for concentrations past the CMC. Furthermore, there is a good agreement between the three-step model and adsorption data from different studies in the literature using zwitterionic surfactants, which indicates the relevance of this model to other systems (Figure 5). It could be used as evidence that micellization is also affecting the surfactant adsorption in those reported cases.

**Figure 5:**
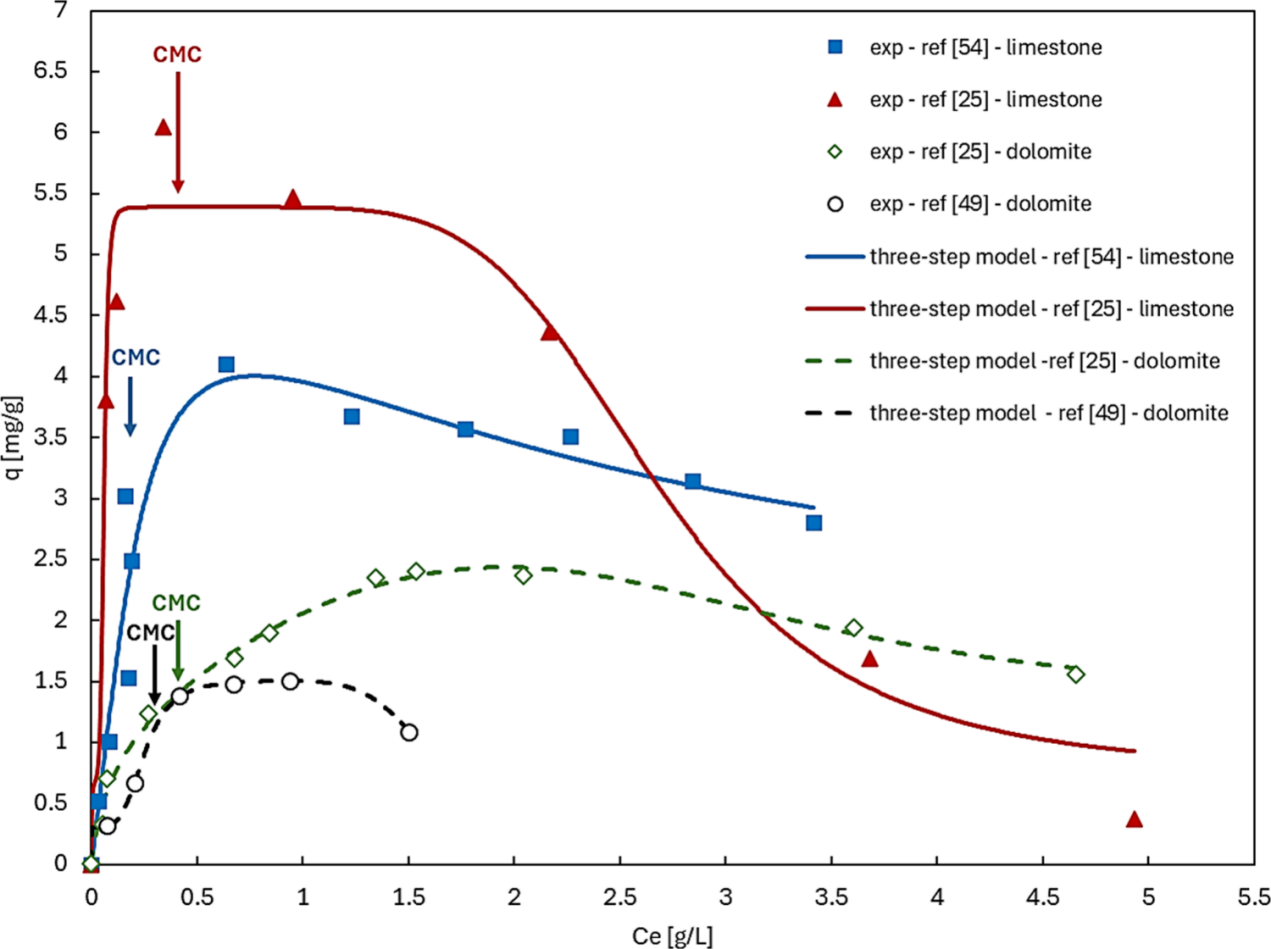
Three-step model applied to literature data on zwitterionic surfactant adsorption on limestone (solid lines) and dolomite (dashed lines). The CMC was adjusted to 300 ppm for the reference^49^ data because it was not demonstrated on their paper. Data represented by triangles and diamonds were reproduced with permission from [25]. Copyright © 2020 Elsevier B.V. Data represented by circles was reproduced with permission from [49]. Copyright © 2024 American Chemical Society. Data represented by squares were reproduced with permission from [54]. Copyright © 2014 American Chemical Society.

The good performance of our model with the literature data (Figure 5), accounting for the CMC as input in each case, suggests that surface aggregates are formed on those mineral surfaces. These aggregates appear to be affected by the presence of micelles, leading to reduced surfactant adsorption beyond the CMC. It can also be hypothesized that interactions occur between micelles and surface aggregates, even if the data appear to indicate only monomer adsorption, as suggested by the good fitting of a modified Langmuir model presented by Nieto-Alvarez and colleagues.^54^

The CMC values are rarely correlated to adsorption data, even though micelles play an important role in adsorption phenomena. As demonstrated by the three-step model, it is crucial to assess this value prior to adsorption experiments, since using it as an input can enhance the accuracy of adsorption prediction. This is particularly important for commercial surfactant formulations, which can contain impurities with interfacial activity, thus modifying the CMC value of the active compound.^55,56^

### Adsorption Maxima in Surfactant Adsorption Isotherms

There are several hypotheses in the literature that can be considered to explain the occurrence of surfactant adsorption maxima in the zwitterionic surfactant isotherms.

#### Intra-Pore Clogging

Pore clogging might happen when the intragrain pore size is smaller than the micelle size. Micelles could block the grain inner surface from being accessed by other monomers, resulting in lower adsorption past the CMC due to a decreasing number of available sites.^57,58^ The average minor and major axis diameters of CAPB micelles are 5.6 and 8.4 nm, respectively.^59^ Since the average equivalent pore diameter of the rocks used in this work, obtained by BJH analysis, was 12 nm for sandstone and 13 nm for limestone, it can be considered that CAPB micelles could go through the adsorbent pores. Therefore, in our case, pore clogging can be dismissed as the main cause for adsorption maxima.

#### Competition with Micelle/Vesicle Equilibrium

This was attributed as a cause of adsorption maxima in previous studies,^25,54^ and it is already incorporated in the three-step model. The major difference is that, in our model, the micelle–monomer equilibrium only affects the hemimicelle desorption step, not the monomer one. If the coefficient *n* gets closer to unity (not equal to the unity), the model approximates to a modified Langmuir isotherm^54^ but still accounts for a degree of surface aggregates getting excluded from the interface.

#### Competitive Adsorption

In the presence of other surfactants with higher surface activity, the main surfactant may desorb in favor of the others. The main surfactant will be affected by the equilibrium concentration of the other surfactants. However, since the more active surfactants adsorb more, their equilibrium concentrations remain low, limiting their impact on the main surfactant’s desorption. Also, it is worth noting that, if competition is solely the reason for adsorption maxima, we lose the discontinuity at the CMC and thus the influence of micelles on the system. If that were the case, it would be common to have adsorption decreasing before the CMC, not after, but this implication is not aligned with observations in the literature and with our data.

#### Impurities

Experiments and theory suggest that the presence of impurities with higher surface activity than the surfactant itself causes the surface tension minimum observed when determining the CMC by tensiometry.^60,61^ This is attributed to their lower solubility in water, which contributes to their solubilization by micelles, thus restoring surface tension.^60,61^ One can think about an analogous mechanism occurring in solid–liquid interfaces as the cause of adsorption maxima, yet highly surface-active impurities getting solubilized by micelles will lead to more available sites, thus more adsorption of the surfactant, contrasting with the surface tension case. The only possibility that this mechanism would generate an adsorption maximum would be if the solubilization of impurities triggers the desorption of surfactants in surface aggregates containing these impurities. Nonetheless, in this case, the adsorption behavior would remain consistent with the three-step model, in which surfactant desorption is mediated by micelles. The difference would be that, in the presence of such impurities, the effect is indirect. If that hypothetical mechanism is true, a pronounced maximum effect (i.e., a high *K*_*m*_ value) would be observed in the adsorption curve of the impurity in the solid–liquid interface. We leave this hypothesis for future studies.

#### Micellar Exclusion

According to Hanna et al., when micellar exclusion is the governing factor, the adsorption maximum is not obtained for surfactants with headgroups oppositely charged to the surface.^58^ In the case of zwitterionic surfactants, since both charges at the headgroup can interact with the mineral surfaces, the occurrence of an adsorption maximum governed by micellar exclusion would be possible.

Given the desorption mechanism in the three-step model proposed in this work, and the good agreement with experimental adsorption data, micelle-induced desorption of surface aggregates mediated by micelle exclusion can be considered the dominant factor responsible for the adsorption maxima observed in CAPB adsorption isotherms. The occurrence of this maximum implies that aggregates interact by the functional headgroup carrying the same charge as the surface sites. In the case of limestone, the effect of micellar exclusion might diminish due to a more heterogeneous charge distributions.^23^

This conclusion can be extended to anionic surfactants, such as sodium dodecyl sulfate (SDS), whose isotherms had shown discontinuities starting at the CMC.^62,63^ Literature data of SDS adsorption on graphite^62^ and carbon black^63^ were used to test the model (Figure 6). The three-step model adequately fits the experimental data, capturing the discontinuity and the maximum adsorption beyond the CMC. In this case, the adsorption maximum can be explained considering the negative nature of the adsorbent surfaces,^64,65^ the induced hemimicelle desorption by micelles, and their consequential exclusion from the interfacial region.

**Figure 6:**
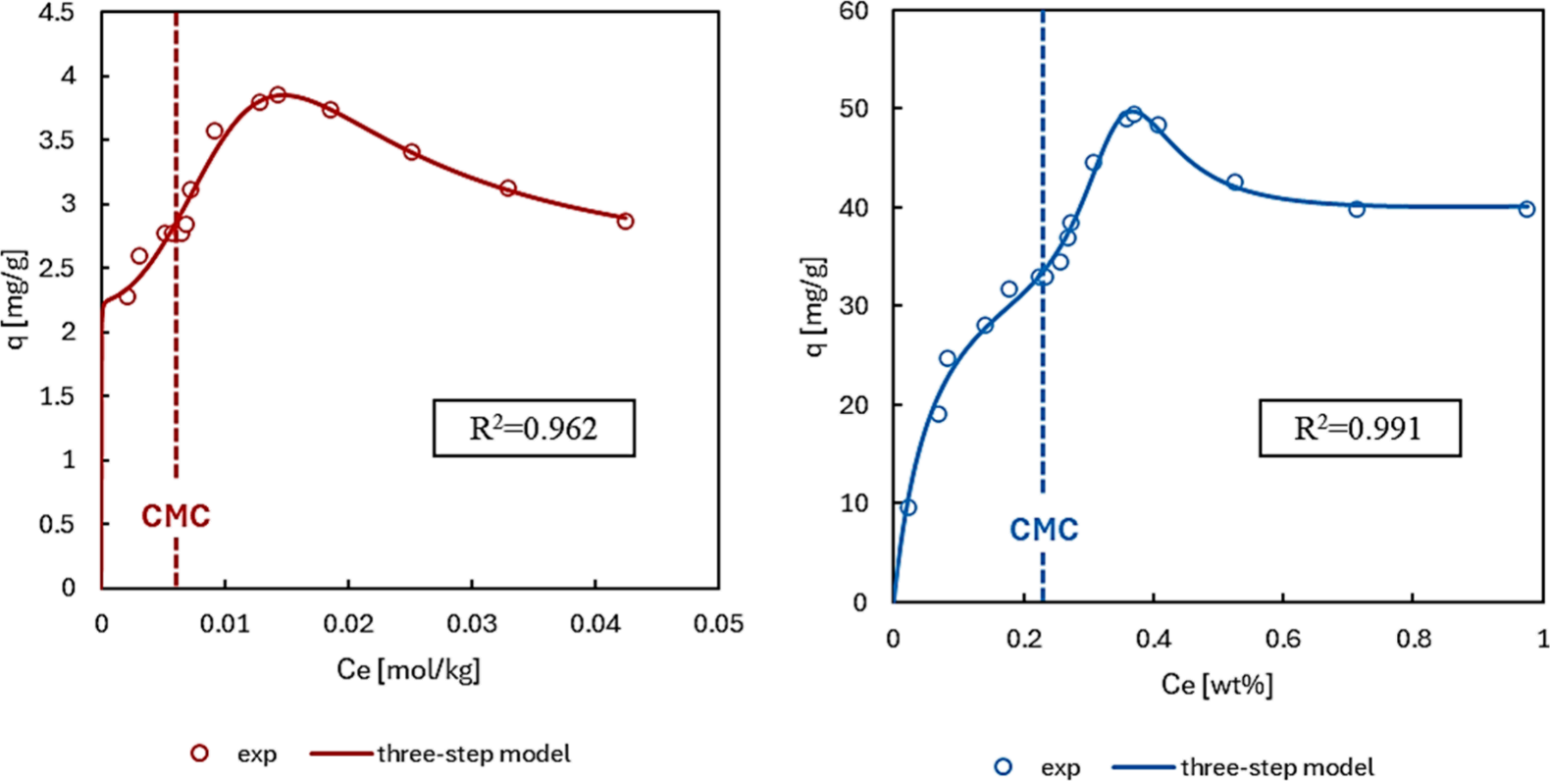
Three-step model applied to literature data on SDS adsorption on graphite^62^ (left) and carbon black^63^ (right). Equilibrium concentration units are maintained as reported in literature. Data on the left were reproduced with permission from [62]. Copyright © 1949 Elsevier Inc. Data on the right were reproduced with permission from [63]. Copyright © American Chemical Society.

By evaluating the model with anionic surfactant data, we show that the three-step model extends to ionic surfactants and their mixtures. Model limitations are governed not only by surfactant type but also by the adsorbent surface charge. If the surface charge density is too low to induce micellar exclusion, the three-step model effectively reduces to the classical two-step model. Therefore, the proposed model is most suited for the adsorption of ionic surfactants when the adsorbent/rock surface carries enough charge to repel the micelle–hemimicelle clusters.

## Conclusions

This work aimed to investigate the mechanisms of CAPB adsorption on sandstone and limestone formations with high salinity conditions, and to support adsorption estimations within high surfactant concentration scenarios. A theoretical-experimental approach was performed to correlate adsorption data with rock surface charges and surfactant aggregation. A general three-step model was developed and formalized as a tool to extend previous theories and address the complexity of surfactant adsorption behavior involving aggregation. In this novel approach, SCM results were correlated to surfactant adsorption data for inferring a new desorption mechanism added to an isotherm model. The results obtained for CAPB adsorption behavior, as interpreted through our newly developed model, lead to the following conclusions:

- CAPB adsorption is more favored on sandstone compared to limestone at concentrations higher than the CMC. This is reinforced by the adjusted equilibrium constants from the three-step model.
- The three-step adsorption model proposed for zwitterionic surfactants is in excellent agreement with our adsorption data and with data from the literature. Its performance is superior to the two-step model, both in explaining and predicting surfactant adsorption behavior on minerals. The three-step model could also be used to predict discontinuous adsorption behavior of other types of ionic surfactants on minerals with concentration intervals including the CMC.
- The micelle-induced desorption of hemimicelles is the governing mechanism generating adsorption maxima for zwitterionic surfactants on both formations. Micellar exclusion effects might diminish due to higher charge heterogeneity in the solid–liquid interface.
- For each surfactant formulation, the CMC should be determined by using a method consistent with the conditions of the adsorption experiments; in our study, pyrene fluorescence was identified as the most suitable technique. Incorporating the experimentally determined CMC value into the three-step model was critical for achieving reliable fits to the discontinuous adsorption behavior observed in our system.

Further investigation needs to be done to derive parameters for the surfactant interactions on the EDL and its precise adsorption influence on zeta potential and surface speciation.

Micelle and hemimicelle dynamics were proven to be important in scenarios of high surfactant concentration, since micelles can dismantle cooperative adsorption and approximate the isotherm to a Langmuir type, or they could not be sufficient to prevent total surface coverage by hemimicelles. These equilibrium scenarios will depend on the relationship between exponents *m* and *n* in the three-step model, as well as the EDL properties, and both must be addressed prior to long-term injection operations using highly concentrated surfactant solutions.

## Supplementary Material



## Data Availability

Data presented in the figures and the MATLAB code used to fit adsorption isotherms may be accessed at https://data.mendeley.com/datasets/f56xt7wt4p/1.

## Notes

Details of CAPB molecular structure and rock sample mineralogy; chromatogram peaks for CAPB identification under isocratic and gradient elution; mathematical deduction of two-step and three-step models with generalized assumptions; pyrene fluorescence spectrum for different CAPB concentrations and I:III peak ratio modeled by Boltzmann regression; fitting scheme implemented in MATLAB; and a detailed description of Surface Complexation Model reactions and parameters and a deduction of the three-step model convergence when concentration approaches infinity for *m* > *n*–1 and *m* < *n*–1 cases (PDF)

## ABBREVIATIONS

CAPB: cocamidopropyl betaine
SCM: surface complexation model
DSW: desulfated seawater
HPLC: high performance liquid chromatography.
