# Supplementary material for: Mechanistic Modeling of Zwitterionic Surfactant Adsorption on Mineral Surfaces: A Three-Step Isotherm with Micelle-Induced Desorption
Source: Langmuir. 2025 Aug 26;41(35):23726–36. doi: 10.1021/acs.langmuir.5c02879 (PMC12424166; doi:10.1021/acs.langmuir.5c02879)
Supplement: Supplementary file 1 [file la5c02879_si_001.pdf]

Mechanistic Modeling of Zwitterionic Surfactant Adsorption on Mineral Surfaces: A Three-Step Isotherm with Micelle-Induced Desorption

Pablo A. Godoy, [a] Luis Maqueira,[b] Aurora Pérez-Gramatges*[a,b]

[a] *Department of Chemistry, Pontifical Catholic University of Rio de Janeiro (PUC-Rio), Rio de Janeiro, Brazil*

[b] *Laboratory of Physical-Chemistry of Surfactants (LASURF), Pontifical Catholic University of Rio de Janeiro (PUC-Rio), Rio de Janeiro, Brazil*

*Corresponding author

Email: aurora@puc-rio.br

Supporting Information

Materials and Methods

Surfactant

The zwitterionic surfactant used in this work is a commercial mixture in which the main component is a C12 carboxy amido betaine.

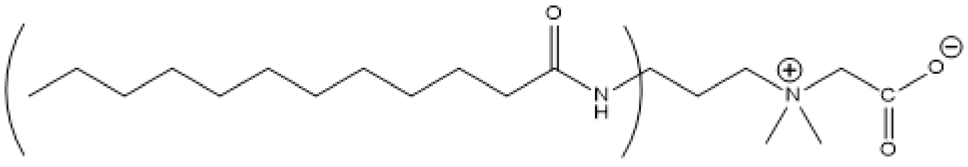

Figure S1. Chemical structure representation of cocamidopropyl betaine (CAPB)

Rock samples

Adsorbent material was made from crushed rock cores from both Berea Sandstone and Indiana Limestone samples, and their mineralogy is described in various sources (including sources in which samples were acquired with the same supplier).

Table S1. Mineral composition of the rocks utilized as adsorbents

Minerals | Rocks	Mineral composition (%)	
	Berea Sandstone[1–4]	Indiana limestone[2,5–8]
Quartz	87.8 ± 2.1	0.6 ± 0.5
Feldspar	5.2 ± 1.5	-
Dolomite	1.1 ± 0.7	0.4 ± 0.5
Kaolinite	4.3 ± 2.2	-
Calcite	-	98.4 ± 1.0

HPLC chromatogram for CAPB

The analytical method was based on gradient elution instead of isocratic, as the identified CAPB peak appears more distant from the matrix peak. This decreases the influence of the matrix components in the peak area utilized for surfactant quantification.

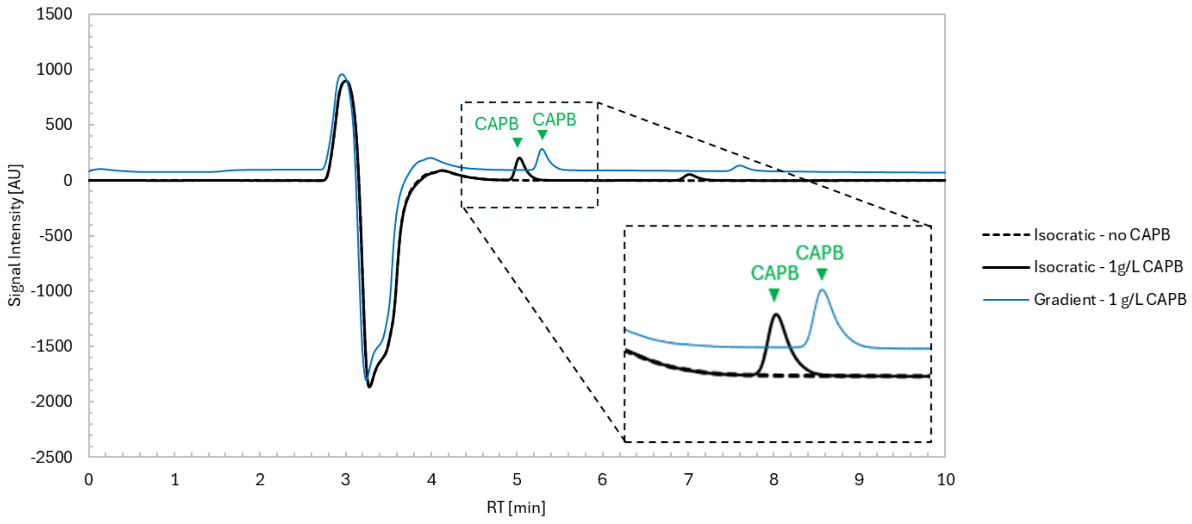

Figure S2. CAPB chromatogram peak with different elution methods

Three-step model based on two-step theory

The adsorption rate equation for the first step (monomer adsorption/desorption), assuming a first-order process, can be expressed as:

$$\frac{d\Gamma_1}{dt} = k_1\Gamma_s C - k_2\Gamma_1 \qquad \text{Eq. S1}$$

Where C is the total surfactant concentration in bulk, Γ_1 is the adsorption density from the first step, Γ_s is the free adsorption site density, and k_1 and k_2 are the monomer adsorption and desorption rate constants, respectively.

For the second step, hemimicelle adsorption velocity can be described as $n-1$ order process related to monomer concentration (n is the number of monomers in one hemimicelle), and first order for the monomer adsorption and hemimicelle desorption:

$$\frac{d\Gamma_h}{dt} = k_3\Gamma_1 C^{n-1} - k_4\Gamma_h \qquad \text{Eq. S2}$$

Where Γ_h is the hemimicelle adsorption density (or second step adsorption density), and k_3 and k_4 are the hemimicelle adsorption and desorption rate constants, respectively.

The velocity of hemimicelles desorption induced by micelle formation can be obtained by Equation S3, where m accounts for the number of surfactants in micellar form capable of inducing the desorption of a single hemimicelle. The term $H(C - CMC)$ is the Heaveside function that implies that the term v_m will be equal to zero if $C < CMC$, and different from zero when $C \geq CMC$.

$$v_m = k_5(C - CMC)^m H(C - CMC)\,\Gamma_h \qquad \text{Eq.S3}$$

Therefore, the complete mass-action model proposed for hemimicelle adsorption includes the micelle-induced hemimicelle desorption, which can be considered as the third step in the mechanism for developing the updated adsorption rate equation (Equation S4).

$$\frac{d\Gamma_h}{dt} = k_3\Gamma_1 C^{n-1} - k_4\Gamma_h - k_5\Gamma_h\,(C - CMC)^m H(C - CMC) \qquad \text{Eq.S4}$$

In equilibrium, both the monomer and hemimicelle adsorption rates approach zero, one can derive the following formulas for Γ_1 and Γ_h, as the terms $(C - CMC)^m$ and the Heaviside function are respectively abbreviated to ΔC^m and $H_{\Delta C}$ for convenience (Equations S5 and S6).

$$\Gamma_1 = K\Gamma_s C \qquad \text{Eq.S5}$$

And

$$\Gamma_h = \frac{K_h C^{n-1}\Gamma_1}{1 + K_m \Delta C^m H_{\Delta C}} \qquad \text{Eq.S6}$$

Where C is the surfactant equilibrium concentration, $K = k_1/k_2$ is the adsorption equilibrium constant of the monomer adsorption step, $K_h = k_3/k_4$ is the hemimicelle adsorption equilibrium constant and $K_m = k_5/k_4$ is the desorption ratio between the effect of micelle and spontaneous desorption of hemimicelles.

As defined by Zhu and Gu, the total adsorption capacity (Γ_∞) depends on a combination of the number of free sites (Γ_s), adsorbed monomers (Γ_1), adsorbed hemimicelles (Γ_h), and the total number of monomers on a hemimicelle (n) in which the following relationship is assumed:

$$\Gamma_\infty = n(\Gamma_s + \Gamma_1 + \Gamma_h) \qquad \text{Eq.S7}$$

Note that the adsorption quantities can only alternate between Γ_s, Γ_1 and Γ_h and not the micelle-like surface aggregates because their lifetime is assumed to be very short. Therefore, the total adsorption capacity is the combined quantity of sites, adsorbed monomers and adsorbed hemimicelles, multiplied by the total amount of possible monomers in one adsorption site (n). Combining Equations S5, S6 and S7 we can derive the first adsorption step Isotherm:

$$\Gamma_1 = \frac{\Gamma_\infty}{n} \frac{KC(1+K_m \Delta C^m H_{\Delta C})}{(1+K_m \Delta C^m H_{\Delta C})(1+KC)+KK_h C^n} \quad \text{Eq.S8}$$

Since the total amount of adsorption depends on the quantity of adsorbed monomers (Γ_1) as follows:

$$\Gamma_t = \Gamma_1 + n\Gamma_h \quad \text{Eq.S9}$$

Substituting equation S6 in S9:

$$\Gamma_t = \Gamma_1 + n \frac{K_h C^{n-1} \Gamma_1}{1+K_m \Delta C^m H_{\Delta C}} \quad \text{Eq.S10}$$

Substituting the term related to Γ_1 in Equation S10 from equation S8, we can derive a general formula for the total adsorption of surfactants for the three-step model with micellar-induced hemimicelle desorption mechanism:

$$\Gamma_t = \frac{\Gamma_\infty}{n} \frac{KC(1+K_m \Delta C^m H_{\Delta C})}{(1+KC)(1+K_m \Delta C^m H_{\Delta C})+KK_h C^n} \left(1 + n \frac{K_h C^{n-1}}{1+K_m \Delta C^m H_{\Delta C}}\right) \quad \text{Eq.S11}$$

The term $\Psi_m = K_m \Delta C^m H_{\Delta C}$ is defined as the micellar-induced desorption contribution, and after simplifying Eq. S11 we obtain:

$$\Gamma_t = \frac{\Gamma_\infty}{n} \frac{KC(1+\Psi_m)+nKK_h C^n}{(1+KC)(1+\Psi_m)+KK_h C^n} \quad \text{Eq.S12}$$

If $\Psi_m = 0$ the three-step formula becomes the two-step model:

$$\Gamma_t = \frac{\Gamma_\infty}{n} \frac{(KC+nKK_h C^n)}{1+KC+KK_h C^n} \quad \text{Eq.S13}$$

CMC determination with pyrene as hydrophobicity probe

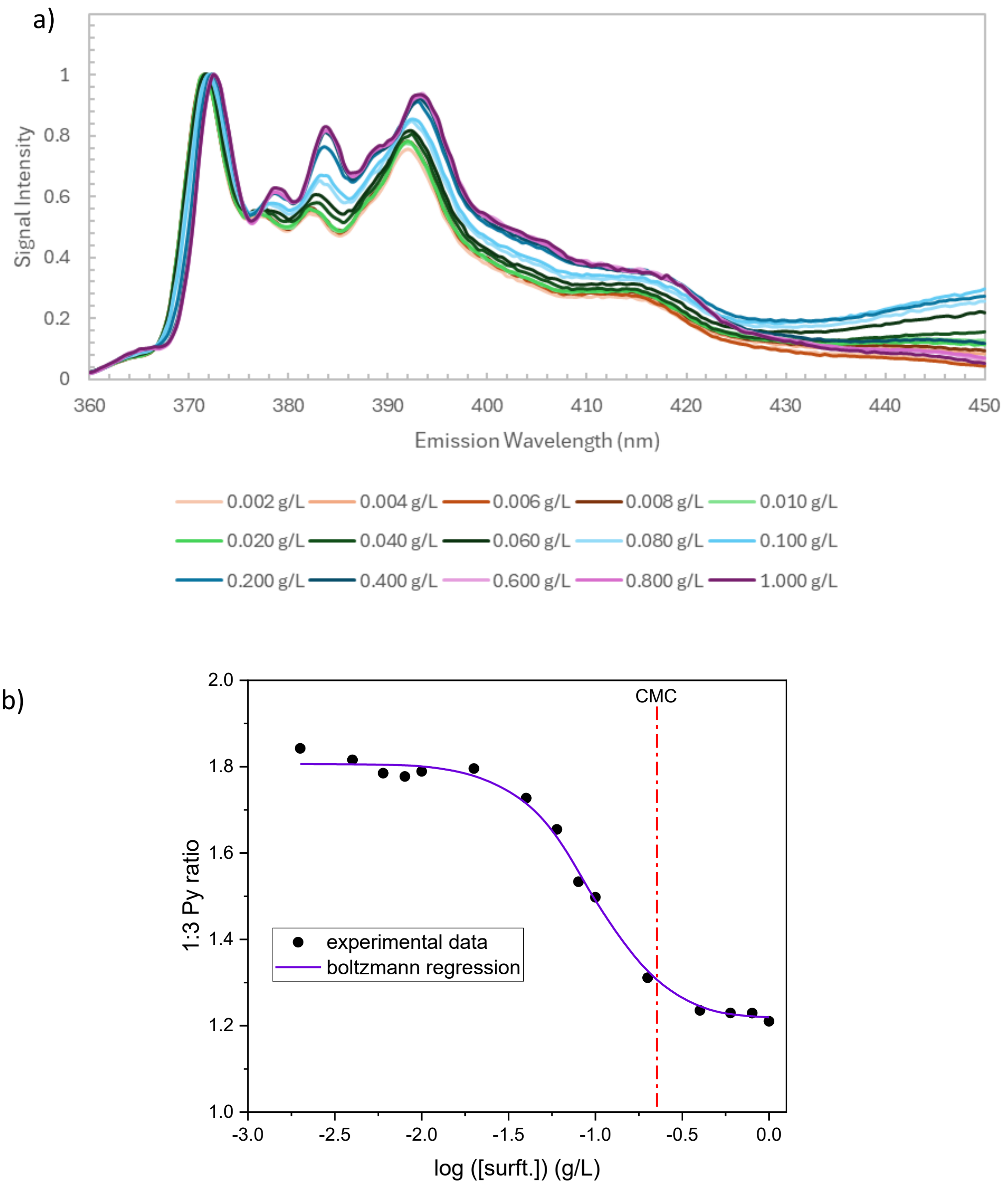

Figure S3. (a) Emission spectrum of pyrene in CAPB solutions, normalized by the maximum intensity and (b) 1:3 pyrene ratio data adjusted by a Boltzmann regression

Critical micelle concentration (CMC) determination with pyrene fluorescence is a consolidated method in the literature that captures very precisely the transition of local hydrophobicity in an aqueous environment when monomers start to form micelles.[9–13] Even though there are multiple approaches for mathematically determining the CMC from raw data, the 1:3 pyrene ratio data, i.e., the ratio between the first and third emission peak heights from pyrene fluorescence obeys a Boltzmann type decreasing sigmoid with extremely accordance.[12] Because of this, the interception of the two lines of each state (unimeric and micellar) can be exactly calculated after obtaining the parameters from a Boltzmann regression (equation S14).

$$y = \frac{A_1 - A_2}{1 + e^{(x - x_0)/\Delta x}} + A_2 \qquad \text{Eq. S14}$$

Adsorption data fitting

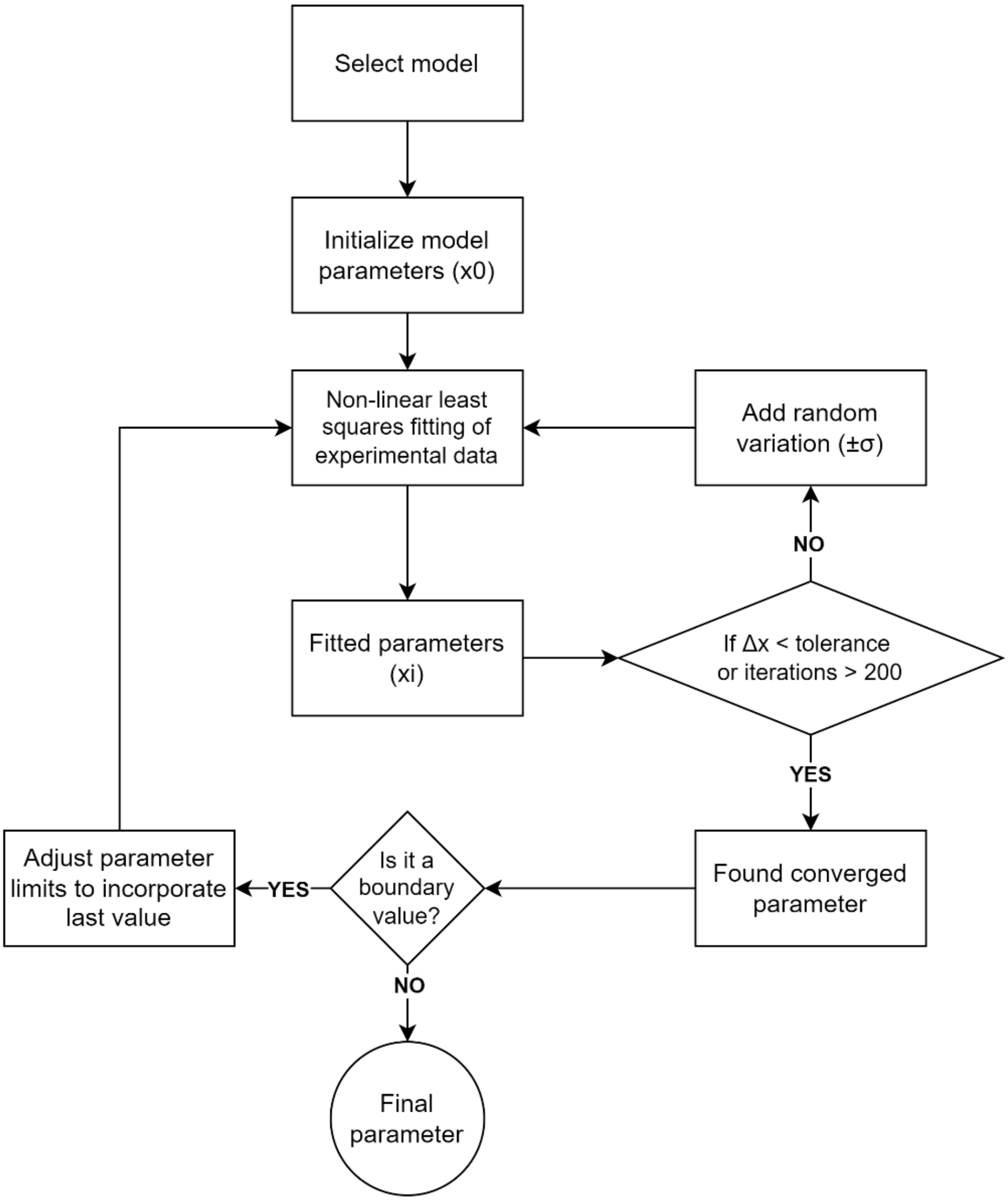

Figure S4. Parameter determination flowchart for isotherm models

SCM reactions and parameters

CD-MUSIC was applied in the works of Takeya et. al[14] and Elakneswaran et. al[1] for explaining both Berea sandstone and calcite zeta potential data and for generating insights into their surface speciation. Their implementation of the model was validated with zeta potential measurements of the mentioned rock and mineral. CD-MUSIC also combines the charge distribution of adsorbed ions in the Stern layers within the Electrical Double Layer (EDL) as well as the existence of multiple surface species in equilibrium with ions in the bulk. The following surface reactions were considered based on the mentioned works:

Table S2. Surface sites considered in each mineral and their reaction equilibrium constants

Reactions	Δz_0	Δz_1	Δz_2	log(K) [25°C]
**Calcite-Brine interface[14]**				
$> CO_3H \rightleftarrows > CO_3^- + H^+$	-1	0	0	-7.3
$> CaOH + H^+ \rightleftarrows > CaOH_2^+$	1	0	0	15
$> CO_3H + Ca^{2+} \rightleftarrows > CO_3Ca^+ + H^+$	-1	2	0	-6.45
$> CO_3H + Mg^{2+} \rightleftarrows > CO_3Mg^+ + H^+$	-1	2	0	-6.15
$> CaOH + H^+ + SO_4^{2-} \rightleftarrows > CaOH_2SO_4^-$	1	-2	0	14.75
**Quartz-Brine interface[1]**				
$> SiOH + H^+ \rightleftarrows > SiOH_2^+$	1	0	0	-1.75
$> SiO^- + H^+ \rightleftarrows > SiOH$	-1	0	0	6.75
$> SiOH + Ca^{2+} \rightleftarrows > SiOCa^+ + H^+$	-1	2	0	-5.70
$> SiOH + Mg^{2+} \rightleftarrows > SiOMg^+ + H^+$	-1	2	0	-5.70
**Kaolinite-Brine interface ($X = Si, Al$)[14]**				
$> XOH + H^+ \rightleftarrows > SiOH_2^+$	1	0	0	0.80
$> XO^- + H^+ \rightleftarrows > SiOH$	-1	0	0	7.00
$> XOH + Ca^{2+} \rightleftarrows > SiOCa^+ + H^+$	-1	2	0	-6.00
$> XOH + Mg^{2+} \rightleftarrows > SiOMg^+ + H^+$	-1	2	0	-5.55

The outer sphere capacitance was calculated for the calcite case, as if the sum of the Stern layers distance was 13Å and the inner sphere length was equivalent to the calcium radius[14]. In the case of the outer sphere capacitance for quartz and kaolinite surface, the values assigned are based on Leroy et. al[15], as the inner capacitance in this case was equivalent to the one assumed for the calcite.

Table S3. Additional parameters to SCM model for each adsorbent

Rock	C1 [F/m²]	C2 [F/m²]	Site / Site density (site/nm²)	
Berea sandstone	3.098	0.2	>SiOH /4.60<br>>XOH /5.55	QUARTZ<br>KAOLINITE
Indiana Limestone	3.098	0.65	>CaOH / 4.95<br>>CO3H / 4.95	CALCITE

Table S4. Site composition of the rocks based on charge

	Surface Species Charge	Fraction
**Sandstone**	Neutral	70.6%
	Negative	15.2%
	Positive	14.1%
**Limestone**	Neutral	1.3%
	Negative	48.7%
	Positive	50.0%

Model convergence limits

Mathematically, we can verify that for $m > n - 1$ the terms with Ψ_m, which comprehends some concentration to the power of m, will grow faster than terms with concentration to the power of $n - 1$. The rearranged equation S11 shows how to approach the limit by dividing the first denominator and numerator for $KC(1 + \Psi_m)$:

$$\lim_{C\to\infty}\Gamma_t = \frac{\Gamma_\infty}{n}\frac{1}{\cancel{\frac{1}{KC}}+1+\cancel{\frac{K_hC^{n-1}}{(1+\Psi_m)}}}\left(1+\cancel{n\frac{K_hC^{n-1}}{1+\Psi_m}}\right) = \frac{\Gamma_\infty}{n}\frac{1}{0+1+0}(1+0) = \frac{\Gamma_\infty}{n}$$

Because $(1 + \Psi_m)$ grows faster than C^{n-1} at higher concentrations the expression would approach the Langmuir equation with a plateau of Γ_∞/n (crossed terms goes to zero), thus as concentration gets high enough, adsorption reaches the Langmuir's limit. This is coherent with the fact that when the plateau approaches Γ_∞/n the surface has one single adsorbed monomer for each site, corresponding with the Langmuir assumption behavior.

When $m < n - 1$ the terms of concentration to the power of $n - 1$ grows faster than the ones to the power of m, and the limit with the rearranged equation S11 (numerator and denominator inside parenthesis multiplied by $1/K_hC^{n-1}$) becomes:

$$\lim_{C\to\infty}\Gamma_t = \frac{\Gamma_\infty}{n}\left(\frac{\cancel{1}}{\frac{\cancel{1}}{\cancel{KC}}+1+\frac{\cancel{K_h C^{n-1}}}{\cancel{(1+\Psi_m)}}} + n\frac{\frac{1}{1+\Psi_m}}{\left(\frac{\cancel{1}}{\cancel{KC}}+1\right)\frac{\cancel{1}}{\cancel{K_h C^{n-1}}}+\frac{K_h C^{n-1}}{(1+\Psi_m)}\frac{1}{K_h C^{n-1}}}\right) =$$

$$= \frac{\Gamma_\infty}{n}\left(0 + n\frac{1/(1+\Psi_m)}{0+1/(1+\Psi_m)}\right) = \frac{\Gamma_\infty}{n}(0+n) = \Gamma_\infty$$

Which is coherent with the fact that there are not enough micelles inducing the desorption of hemimicelles to dismantle surface aggregates, thus leading to more surface coverage until it reaches the maximum capacity for the adsorbent (Γ_∞).

References

(1) Elakneswaran, Y.; Ubaidah, A.; Takeya, M.; Shimokawara, M.; Okano, H. Effect of Electrokinetics and Thermodynamic Equilibrium on Low-Salinity Water Flooding for Enhanced Oil Recovery in Sandstone Reservoirs. ACS Omega 2021, 6 (5), 3727–3735. DOI: 10.1021/acsomega.0c05332.

(2) Churcher, P. L.; French, P. R. Rock Properties of Berea Sandstone, Baker Dolomite, and Indiana Limestone. SPE International Symposium on Oilfield Chemistry 1991. DOI: 10.2118/21044-MS

(3) Van Den Abeele, K. E. -A.; Carmeliet, J.; Johnson, P. A.; Zinszner, B. Influence of Water Saturation on the Nonlinear Elastic Mesoscopic Response in Earth Materials and the Implications to the Mechanism of Nonlinearity. J.-Geophys.-Res. 2002, 107 (B6). DOI: 10.1029/2001JB000368

(4) Afrough, A.; Zamiri, M. S.; Romero-Zerón, L.; Balcom, B. J. Magnetic-Resonance Imaging of Fines Migration in Berea Sandstone. Soc. Pet. Eng. J. 2017, 22 (05), 1385–1392. DOI: 10.2118/186089-PA

(5) Freire-Gormaly, M.; Ellis, J. S.; MacLean, H. L.; Bazylak, A. Pore Structure Characterization of Indiana Limestone and Pink Dolomite from Pore Network Reconstructions. *Oil Gas Sci. Technol. – Rev. IFP Energies nouvelles* **2016**, *71* (3), 33. DOI: 10.2516/ogst/2015004

(6) America, I. L. I. of. *Indiana Limestone Handbook*; Indiana Limestone Institute of America.

(7) Eliebid, M.; Mahmoud, M.; Shawabkeh, R.; Elkatatny, S. Surfactants Impact on CO2 Sequestration for Enhanced Gas Recovery and in Depleted Carbonate Reservoirs. Abu Dhabi International Petroleum Exhibition & Conference 2017. DOI: 10.2118/188687-MS

(8) Lucas, C. R. D. S.; Aum, Y. K. P. G.; Araújo, E. D. A.; Castro Dantas, T. N. D.; Araújo, E. A.; Sousa, T. N.; Aum, P. T. P. Investigating the Fluid–Solid Interaction of Acid Nonionic Nanoemulsion with Carbonate Porous Media. *Molecules* **2020**, *25* (6), 1475. DOI: 10.3390/molecules25061475.

(9) Kalyanasundaram, K.; Thomas, J. K. Environmental Effects on Vibronic Band Intensities in Pyrene Monomer Fluorescence and Their Application in Studies of Micellar Systems. *J. Am. Chem. Soc.* **1977**, *99* (7), 2039–2044. DOI: 10.1021/ja00449a004.
(10) Glushko, V.; Thaler, M. S. R.; Karp, C. D. Pyrene Fluorescence Fine Structure as a Polarity Probe of Hydrophobic Regions: Behavior in Model Solvents. *Arch. Biochem. Biophys.* **1981**, *210* (1), 33–42. DOI: 10.1016/0003-9861(81)90160-0.
(11) Alargova, R. G.; Kochijashky, I. I.; Sierra, M. L.; Kwetkat, K.; Zana, R. Mixed Micellization of Dimeric (Gemini) Surfactants and Conventional Surfactants. *J. Colloid Interface Sci.* **2001**, *235* (1), 119–129. DOI: 10.1006/jcis.2000.7311.
(12) Aguiar, J.; Carpena, P.; Molina-Bolívar, J. A.; Carnero Ruiz, C. On the Determination of the Critical Micelle Concentration by the Pyrene 1:3 Ratio Method. *J. Colloid Interface Sci.* **2003**, *258* (1), 116–122. DOI: 10.1016/S0021-9797(02)00082-6.
(13) Piñeiro, L.; Novo, M.; Al-Soufi, W. Fluorescence Emission of Pyrene in Surfactant Solutions. *Adv.Colloid Interface Sci.* **2015**, *215*, 1–12. DOI: 10.1016/j.cis.2014.10.010.
(14) Takeya, M.; Ubaidah, A.; Shimokawara, M.; Okano, H.; Nawa, T.; Elakneswaran, Y. Crude Oil/Brine/Rock Interface in Low Salinity Waterflooding: Experiments, Triple-Layer Surface Complexation Model, and DLVO Theory. *J. Petrol. Sci. Eng.* **2020**, *188*, 106913. DOI: 10.1016/j.petrol.2020.106913.
(15) Leroy, P.; Revil, A. A Triple-Layer Model of the Surface Electrochemical Properties of Clay Minerals. *J. Colloid Interface Sci.* **2004**, *270* (2), 371–380. DOI: 10.1016/j.jcis.2003.08.007.
